# Automated Characterization of Cyclic Alternating Pattern Using Wavelet-Based Features and Ensemble Learning Techniques with EEG Signals

**DOI:** 10.3390/diagnostics11081380

**Published:** 2021-07-30

**Authors:** Manish Sharma, Virendra Patel, Jainendra Tiwari, U. Rajendra Acharya

**Affiliations:** 1 Department of Electrical and Computer Science Engineering, Institute of Infrastructure, Technology, Research and Management (IITRAM), Ahmedabad 380026, India; virendra.patel.17e@iitram.ac.in (V.P.); jainendra.tiwari.17e@iitram.ac.in (J.T.); 2 School of Engineering, Ngee Ann Polytechnic, Singapore 599489, Singapore; aru@np.edu.sg; 3 Department of Bioinformatics and Medical Engineering, Asia University, Taichung 41354, Taiwan; 4 School of Management and Enterprise, University of Southern Queensland, Springfield 4300, Australia

**Keywords:** sleep, sleep macrostructure, sleep microstructure, electroencephalogram (EEG), polysomnogram (PSG), cyclic alternating pattern (CAP), classification, ensemble of bagged trees (EBagT)

## Abstract

Sleep is highly essential for maintaining metabolism of the body and mental balance for increased productivity and concentration. Often, sleep is analyzed using macrostructure sleep stages which alone cannot provide information about the functional structure and stability of sleep. The cyclic alternating pattern (CAP) is a physiological recurring electroencephalogram (EEG) activity occurring in the brain during sleep and captures microstructure of the sleep and can be used to identify sleep instability. The CAP can also be associated with various sleep-related pathologies, and can be useful in identifying various sleep disorders. Conventionally, sleep is analyzed using polysomnogram (PSG) in various sleep laboratories by trained physicians and medical practitioners. However, PSG-based manual sleep analysis by trained medical practitioners is onerous, tedious and unfavourable for patients. Hence, a computerized, simple and patient convenient system is highly desirable for monitoring and analysis of sleep. In this study, we have proposed a system for automated identification of CAP phase-A and phase-B. To accomplish the task, we have utilized the openly accessible CAP sleep database. The study is performed using two single-channel EEG modalities and their combination. The model is developed using EEG signals of healthy subjects as well as patients suffering from six different sleep disorders namely nocturnal frontal lobe epilepsy (NFLE), sleep-disordered breathing (SDB), narcolepsy, periodic leg movement disorder (PLM), insomnia and rapid eye movement behavior disorder (RBD) subjects. An optimal orthogonal wavelet filter bank is used to perform the wavelet decomposition and subsequently, entropy and Hjorth parameters are extracted from the decomposed coefficients. The extracted features have been applied to different machine learning algorithms. The best performance is obtained using ensemble of bagged tress (EBagT) classifier. The proposed method has obtained the average classification accuracy of 84%, 83%, 81%, 78%, 77%, 76% and 72% for NFLE, healthy, SDB, narcolepsy, PLM, insomnia and RBD subjects, respectively in discriminating phases A and B using a balanced database. Our developed model yielded an average accuracy of 78% when all 77 subjects including healthy and sleep disordered patients are considered. Our proposed system can assist the sleep specialists in an automated and efficient analysis of sleep using sleep microstructure.

## 1. Introduction

Sleep is an important aspect of human life and it greatly affects our mental and physical health. Sleep consists of periodic repetition of unconsciousness (physical-inactivity) called non rapid eye moments (NREM)) followed by high activity called rapid eye moments (REM). The REM and NREM manifests certain important functioning of brain including memory consolidation, brain clearance from metabolites and cellular restoration. However, the entire process is yet not completely perceived and known. To distinguish sleep’s macrostructure, sleep is categorized into five stages: wakefulness (W), N1, N2, N3, and REM, according to the guidelines of American Academy of Sleep Medicine (AASM) [1]. The distinction is made on the 30 s window of electroencephalogram (EEG) signal by the trained medical practitioners. The sleep stages N1, N2, and N3 form NREM part of the sleep cycle followed by REM. In literature, many studies are available on the macrostructure of sleep and researchers have developed models for automated classification of sleep stages using machine learning techniques and PSG [2,3,4,5,6,7,8,9]. Recently, deep learning-based methods have also been employed for sleep scoring [10,11,12].

However, ephemeral events such as K-complexes and transient power alterations in frequency bands are neglected by these macrostructure based sleep scoring rules. In the AASM guidelines, only the arousal definition captures the short periods of changes in cortical activation. Although, phasic events like K-complexes and delta bursts show characteristics similar to arousal, but they are not considered as arousal if there is no to short-term frequency increase in EEG [13,14]. To overcome these shortcomings of macrostructure based sleep scoring, a new microstructure based sleep scoring technique named cyclic alternating pattern (CAP) was devised, which includes such phasic events in brain activity [15] as an alternative scheme to describe NREM sleep. CAP is found to be useful in the detection of insomnia, sleep apnea syndrome, epileptic disorders and periodic limb movements [16]. Ferini-Strambi et al. have observed the impact of CAP on heart rate variability during sleep in healthy young adults [17]. They concluded that, the cardiac autonomy in normal subjects is influenced by the physiological fluctuations of EEG arousal level. Studies have found that during coma, the CAP in EEG signal is correlated with the motor activity, cardiorespiratory rate and cerebrospinal fluid pressure. These events were observed to increase during phase A and decrease during phase B [18,19].

**Figure 1:**
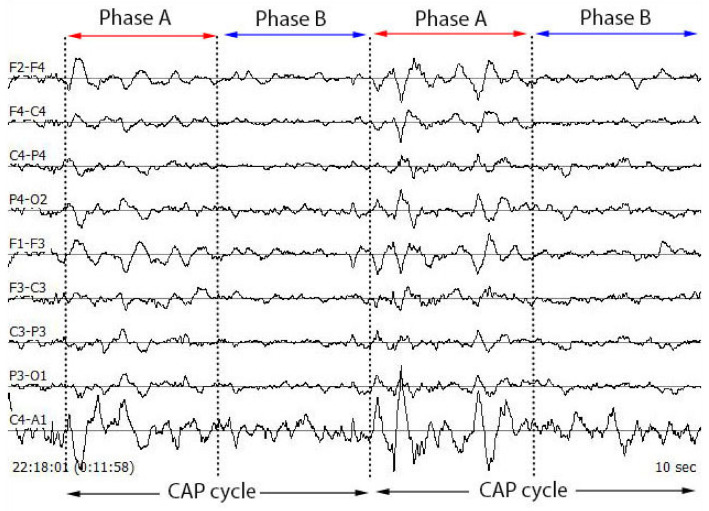
Typical multi-channel image of a CAP cycle with phase A and phase B.

**Figure 2:**
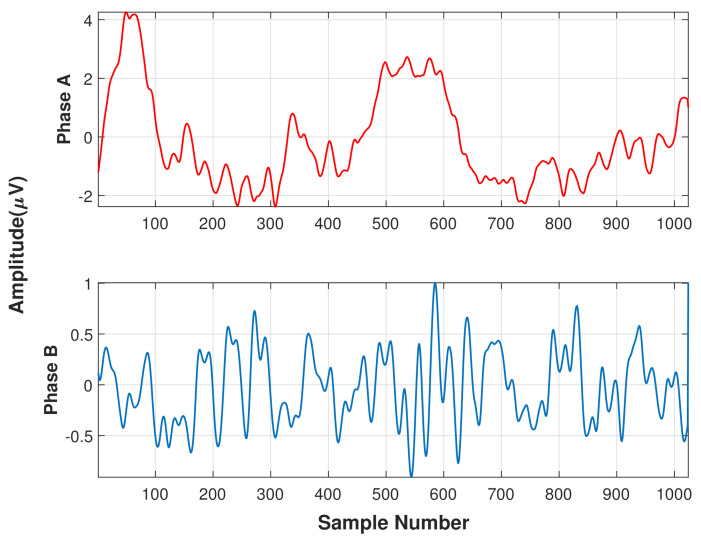
Typical waveforms of Phase A (**top**) and Phase B (**bottom**).

The NREM sleep stage is observed to have alternating patterns of cerebral activation (phase A) followed by duration of deactivation (phase B), which separate two or more consecutive phase A periods [15]. The CAP phase A typically includes events like K-alpha, K-complex sequences, delta bursts, alpha waves, vertex sharp transients and arousals. The duration of these phases may vary between 2 s to 60 s. Two successive phase A events are considered as a single phase A event if the duration of separation between them is less than 2 s [20]. The combination of phase A and phase B is termed as a CAP cycle and this cycle begins with phase A and ends with phase B. Two successive CAP cycles are needed to form a CAP sequence [21]. If a phase A is not accompanied by phase B, then it is termed as an isolated phase A and is considered as non-CAP (an absence of CAP for >60 s duration). Thus, a CAP sequence contains minimum three A phases (A–B–A–B–A) followed by non-CAP period [22]. However, there is no upper limit in terms of overall duration or number of CAP cycles, and approximate mean duration of a CAP sequence in healthy young adults is around 150 s containing six CAP cycles [23]. In general, a CAP sequence always follows a continuous NREM sleep EEG pattern with a minimum duration of 60 s. CAP phase A can be detected using any EEG lead [22]. Figure 1 shows the typical image of a CAP cycle for multiple EEG channels and Figure 2 displays the typical waveforms of phase A and phase B.

High amplitude slow EEG waves increases with increasing depth of sleep whereas low amplitude fast rhythms are dominantly present in REM sleep. CAP phase A is subdivided into three subtypes A1, A2, and A3 based on the duration of high amplitude slow waves and low amplitude fast rhythms. Subtype A1 is characterised by high amplitude slow waves covering >80% of entire phase A duration and low amplitude fast rhythms, if present, covers <20% of total phase A duration. Subtype A2 is characterised by a mixture of fast and slow EEG waves. Low amplitude slow waves in phase A2 covers around 20–50% of entire phase A duration. Subtype A3 is characterised by dominant low amplitude fast rhythms covering >50% of the phase A duration. CAP sequences triggered or interrupted by body movements are also distinguished as subtype A3 [22]. The CAP parameters include CAP rate, CAP time, and no of phases A1, A2 and per hour. Cap rate refers to the percentage ratio of CAP time to total NREM sleep time. CAP time refers to the total duration of all CAP sequences. CAP time increases with increase in number of CAP cycles. For healthy sleepers, CAP rate has very low variability. It is observed to vary with age. It is very low for toddlers (around 13%), gradually increases with age and peaks during peripubertal stage (around 62%), then again decreases for adults and middle age (around 37%) followed by an increase during elderly age (55%) [22].

Thus, the detection of CAP phases and estimation of CAP parameters is essential for accurate sleep analysis. However, the CAP detection in human beings is prone to errors and a cumbersome task. In literature, few attempts have been made for identifying CAP phases automatically using computer aided systems [24,25,26,27,28]. However, the studies on the automated detection of sleep phasic events are very few. Also, the model developed using machine learning methods on the above studies have been tested on very small number (5–10) of good sleepers without considering sleep disordered patients. Further, despite using large number of discriminating features the classification performance is not very high. Hence, there is a need for a model to be tested with significant number of subjects, involving both healthy controls and sleep disordered patients which can exhibit high performance. It is also desirable that the model should employ small number of features for training and testing so that it can be implemented in real time application.

In the proposed study, we have used a large number of subjects comprising of healthy as well as sleep disordered-patients suffering from six different disorders. We have implemented an ensemble learning method by employing wavelet-based Hjorth and entropy features extracted from monopolar C4-A1 and bipolar F4-C4 EEG channels to develop an automated model for detection of CAP phases. The developed model achieved better classification performance than the existing state-of-art studies. Our developed method is simple, computationally efficient and hence it may be deployed in clinical applications.

## 2. Material Used

This study is accomplished using the public CAP sleep database, which contains night-long sleep polysomnographic (PSG) recordings of 108 subjects logged at the Sleep Disorders Center of the Ospedale Maggiore of Parma, Italy. The database contains recordings of 16 healthy subjects and patients suffering from insomnia (9), narcolepsy (5), nocturnal frontal lobe epilepsy (40), periodic leg movement (10), REM behavious disorder (22), and sleep-disordered breathing(4). Each PSG recording contains multiple channels including EEG, electrocardiogram (ECG), electromyogram (EMG), electrooculogram (EOG), SpO2 and respiratory signals. EEG channels include traces like F4-C4, C4-A1, C4-P4, P4-O2, Fp2-F4, Fp1-F3, C3-P3, P3-O1 and other combinations of F3/F4/C3/C4/O1/O2 with reference A1/A2. The sampling frequencies of these EEG channels varies from 100 Hz to 512 Hz. Bipolar EEG channel F4-C4 and monopolar EEG channel C4-A1 are present in maximum number of sleep recordings. Based on the availability of C4-A1 and F4-C4 channels coupled with 512 Hz sampling frequency, we have taken 77 subjects for the identification of CAP phase A in this study. Terzano et al. [21] suggested that bipolar EEG leads are favourable for CAP phase detection. So we have also evaluated performance of each of the two leads (F4-C4 and C4-A1) independently and combined. We have performed CAP phase classification using EEG signals from healthy subjects as well as disordered patients individually and combined. Table 1 shows gender and age details of healthy controls and sleep disordered patients used in this study.

## 3. Methodology

**Figure 3:**
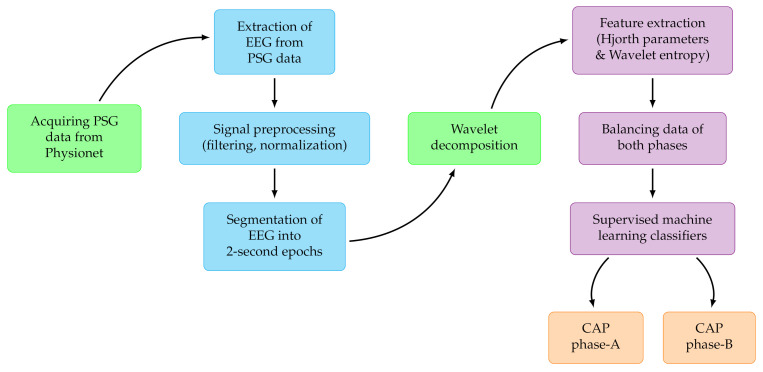
Flow diagram of the proposed study.

Figure 3 represents the procedure involved in the automated identification of CAP phase A and phase B. Firstly, we collected PSG recordings of all 77 subjects then EEG recordings are extracted with sampling frequency of 512 Hz. We separated C4-A1 (monopolar) and F4-C4 (bipolar) EEG montages for this study. Both EEG channels are reprocessed via normalization and filtering operations. Subsequently, labelled A and B phases are segmented using windows of one and two seconds duration. Each EEG epoch (of lengths either 1 s or 2 s) is subjected to an optimal mean squared bandwidth minimized (OMSBM) orthogonal wavelet filter bank (OWFB) that decomposed the EEG epochs into six subbands [29]. The wavelet entropy and Hjorth parameters of each subband are computed from the decomposed subband coefficients and used as discriminating features. We have employed several machine learning classifiers to distinguish the features to choose the optimum performing classifier. Ten-fold cross-validation is performed to develop the model.

### 3.1. Preprocessing

EEG signals contains most of the useful information in the frequency range below 35 Hz. Hence, to remove the noise artifacts and preserve only the necessary information, EEG signals are bandpass filtered using an infinite impulse response (IIR), butterworth filter of order four [30,31]. The lower and upprer cut-off frequencies have been set to 0.5 Hz and 35 Hz, respectively as mentioned in guidelines of AASM [1]. After filtering, normalization of the signal is done to uniformly scale down the amplitude in the range of 0 to 1.

CAP sleep database contains phase A annotations scored by expert neurologists in accordance with the Terzano’s reference atlas of rules [15]. With the help of the annotations, we have separated CAP phase A recording from the NREM sleep EEG signal and labelled remaining NREM signal as non-CAP recording. These phase A (CAP) and phase B (non-CAP) signals are segmented into epochs of 2 s duration each. Later, in order to develop a precise CAP scoring system, we have also obtained CAP classification results using 1 s duration epoch of each phase. All EEG epochs are balanced with respect to both phase A and phase B. For balancing we used random undersampling to reduce the number of epochs from either phase to equalize epoch count of both phases.

### 3.2. Orthogonal Filter Bank and Wavelet Decomposition

There are many wavelet filter banks available in the literature with wide variety of applications [32,33,34,35,36]. Among these filter banks, the orthogonal filter bank is remarkable due to its advantage of energy preservation [37,38,39]. In this study, we have used the orthogonal wavelet filter with minimum root-mean-square (RMS) bandwidth [40,41,42]. Generally, equiripple filter method and band-energy minimization method are used to achieve sharper roll-off. Although, these methods are efficient, but they do not take entire spectrum into account as they are based on edge frequency specifications. On the other hand, RMS bandwith simultaneously considers both transition band and pass/stopband, comprehensively describing a filter’s frequency localization and avoiding influence of ripple amplitude specifications [43,44]. In this method, for the optimization of RMS bandwidth, either a symbolic method can directly applied [40] or constrained optimization problem that is transformable into a convex optimization problem like semidefinite programming (SDP) problem [45] can be used. The SDP includes non-negativity constraints during the optimization. We have used orthogonal filter with length 12 and 4 vanishing moments [46] in this study. The significance of this method is that, it can give more accurate and thorough results including all local and global minimum [47,48].

**Figure 4:**
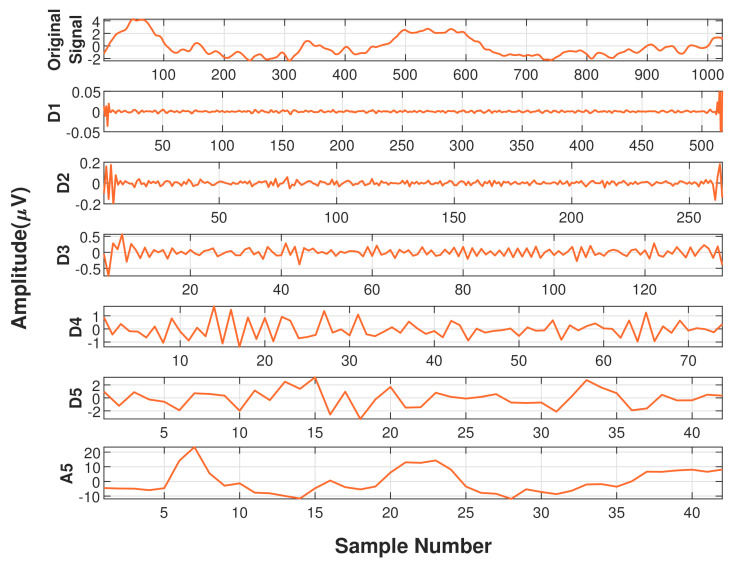
Subbands obtained after wavelet decomposition of a 2-second epoch of CAP Phase-A.

**Figure 5:**
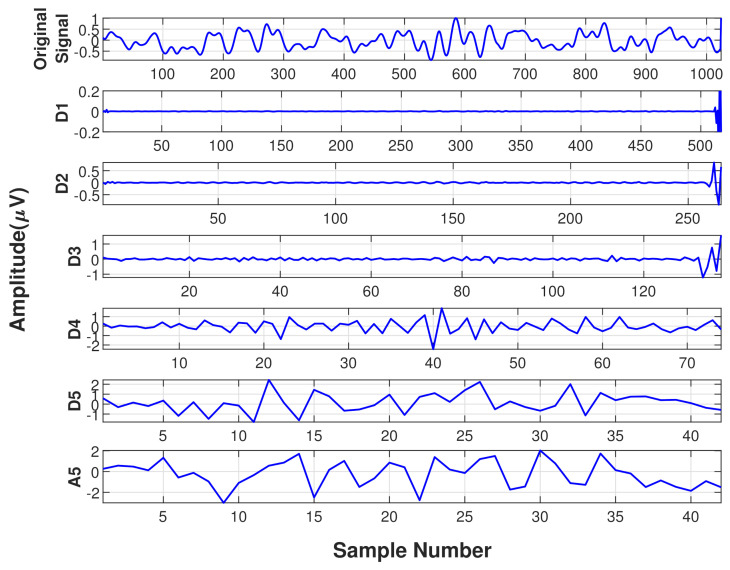
Subbands obtained after wavelet decomposition of a 2-second epoch of CAP Phase-B.

Five level wavelet decomposition yielded six subbands with frequency ranges 0–1 Hz, 1–2 Hz, 2–4 Hz, 4–8 Hz, 8–16 Hz, 16–32 Hz. In this work, lower frequency band (0–1 Hz) is called the approximation coefficient and remaining bands with higher frequencies are called detailed coefficients [49]. Figure 4 and Figure 5 displays the subbands after wavelet decomposition for an epoch of phase A and phase B, respectively.

### 3.3. Feature Extraction

We computed wavelet entropy and three Hjorth parameters from each subband to get 48 discriminating features for CAP phase identification using two EEG channels. The size of the feature array employed is 165960×48. The details of discriminating features are as follows:

**Wavelet Entropy:** Wavelet entropy is used for quantitative analysis of transient features of non-stationary physiological signals including EEG. It is able to measure the uncertainty involved in a random process and for a wavelet subband it can be defined as [50]:

(1) $$E_{Wave}=-\sum_{i=1}^{n}x_{i}log\left(x_{i}\right)$$

where, $x_{i}$ represents the magnitude of $i^{th}$ wavelet coefficient.

**Hjorth Parameters:** Three Hjorth parameters named activity, mobility and complexity are utilized in our study along with wavelet entropy for feature extraction which are extensively used in applications related to EEG signals. These parameters mainly represent the time domain characteristics of EEG signals [51]. Table 2 provides the description of these parameters and their expression. In order to measure, discriminating abilities of each feature, we have computed the *p*-values and rank of each feature using Kruskal-Wallis test. Table 3 shows the *p*-values and ranks for all 48 features computed using Kruskal-Wallis test. From the table, it is clear that p-vales of all features are close to zero. Hence, all features are statistically significant for the classification task.

### 3.4. Classification and Validation

After performing feature extraction, features have been applied to several supervised machine learning (ML) classifiers. For the implementation of these algorithms and to develop the model, we have employed the Statistics and Machine Learning Toolbox of MATLAB R2020a. The obtained features are used to train various classifiers namely support vector machines (SVM), K-nearest neighbour (KNN), ensemble bagged trees (EBagT) and ensemble boosted trees (EBoosT). We developed the model using sample-wise ten-fold cross-validation scheme. Initially, all classifiers are trained using a trial and error approach, because it is difficult to determine in advance which algorithm will perform better for our extracted features. Once the best classier is obtained among all, we performed hyperparameter tuning to increase the performance further. We observed that, for most of the classification tasks EBagT and EBoosT algorithms attained the best classification performance after extensive simulations, however, for few classification tasks SVM algorithm showed better results. It can be noted from Table 1 that, we have considered large number of epochs in our study and ensemble methods yielded better results compared to other algorithms. It is to be noted that we have balanced the epochs of both phases before training the classifiers. The data balancing makes the model more reliable for practical applications.

## 4. Results

The entire experimentation and training related to our study was accomplished using MATLAB R2020a installed on an Ubuntu server 18.04. The specifications of the server are: an Intel Xeon E5-2690 v3 CPU @2.6 GHz (6 cores), 56 GB RAM and a 12 GB Nvidia K80 GPU.

In this work, we have used monopolar C4-A1 and bipolar F4-C4 EEG channels for the identification of CAP phases A and B using sleep recordings of 77 subjects. The database contains recordings of healthy subjects and variety of sleep disorders like NFLE, SDB, narcolepsy, PLM, insomnia and RBD. In this work, we have extracted and analyzed CAP phase information for each type sleep disorders separately. The results are given below for both EEG montages individually and combined. The classification performance was evaluated in terms of average classification accuracy (ACA), precision (Pcn), Recall (Rcl), F1-score (F1), Cohen’s Kappa (κ) value and area under curve (AUC).

We have taken six healthy subjects and extracted 9930 epochs (2 s duration) of phase A and phase B for two EEG channels. Though features have been applied to several classifiers, the optimal performance was obtained using ensemble boosted trees and bagged trees classifier to classify CAP phases. The model could attain ACA of 75.7% and 76.4% for C4-A1 and F4-C4 channels, respectively. It is evident that bipolar channnel F4-C4 channel performed better than the monopolar counter part. Combining the features extracted from both channels yielded better ACA of 83.30%. The confusion matrix corresponding to three classification tasks for good sleepers can be seen in Table 4. It can be observed from Table 4 that, for healthy sleepers both phases are detected equally well while features of both channels are classified together.

The CAP sleep database contains seven EEG recordings for both montages, at sampling rate of 512 Hz with average duration of 575 min, corresponding to insomniac patients. We have classified total of 11328 epochs (2 s) into phase A and phase B. We have achieved maximum ACA of 71.4% and 72.5% for C4-A1 and F4-C4 channels, respectively. It can be seen that bipolar channel F4-C4 performed better than the monopolar channel C4-A1, individually. By combining both EEG channels, ACA increased to 76.5%. Confusion matrix and performance parameters for insomnia subjects are shown in Table 5.

The CAP sleep database contains EEG recordings of five narcolepsy patients, with both montages sampled at 512 Hz frequency and an average duration of 494 min. We have taken 10086 epochs (2 s each) of phase A and phase B. The best possible ACA achieved is 77.20%. The confusion matrix for all three classification tasks can be seen in Table 6.

The CAP database contains 27 EEG recordings of NFLE patients at sampling frequency of 512 Hz and an average duration of 505 min. We have classified 73236 epochs (2 s duration) into phase A and phase B. Among all classifiers, ensemble bagged trees classifier gave best performance with maximum ACA of 84%, F1-score of 0.84 and Cohen’s κ coefficient of 0.68 using both channels. Confusion matrix and performance parameters corresponding to classification using C4-A1, F4-C4 and combination of both can be seen in Table 7.

The CAP database contains nine PLM patients’ EEG recordings for both the montages, at sampling rate of 512 Hz and with an average duration of 431 min. The maximum ACA, F1-score and Cohen’s κ obtained are 77%, 0.76 and 0.54, respectively. Table 8 shows the confusion matrix and performance parameters for patients suffering from PLM.

22 RBD patients’ EEG recordings obtained from CAP sleep database were segmented into total number of 39654 epochs of 2s which contains either phase A or phase B. The average duration for each of these recordings is 514 min. For classifying the phases of RBD patients, the proposed model attained the best ACA of 72%, 70%, and 77% for F4-C4 channel, C4-A1 channel and combined channels, respectively. The F1-score and Cohen’s κ of 0.71 and 0.44, respectively were obtained corresponding to the features obtained from the combined channels. Table 9 shows results obtained using RBD patients.

The CAP sleep database contains one SDB patient’s EEG recording for both the channels, with sampling rate of 512 Hz and 396 min duration. We segregated EEG signals into 1668 epochs (2 s each) which comprises of phases A and phase B. Using EBagT classifier on the extracted features, with C4-A1 and F4-C4 channels separately, the model attained ACA of 74.9% with 10 fold cross validation, whereas combined features from both channels produced ACA of 81.5%. We have obtained F1-score and Cohen’s κ equal to 0.81 and 0.63, respectively for combined features. Confusion matrix and performance parameters obtained for SDB patients are shown in Table 10.

After considering detection of phases of all sleep disordered patients and healthy controls individually, we then combined all 77 subjects with a total of 165,960 epochs corresponding to phases A and B. The model attained ACA of 71.4% and 71.0% with C4-A1 and F4-C4 channels, respectively using ensemble bagged trees classifier with 10 fold CV. On combining features obtained from both channels, ACA increased to 78.0% and F1-score and Cohen’s kappa obtained are 0.77 and 0.56, respectively. It is also interesting to note that both the channels performed equally well in identifying phases when used separately. However, on combining the features from both montages there is a gain of around 7% in the ACA. It can be noticed from the results that, for narcolepsy, NFLE and PLM patients, C4-A1 channel performed better than F4-C4. F4-C4 channel performed better for healthy, insomnia and RBD patients. Both the channels performed almost the same for SDB patients. Table 11 shows the classification results when all subjects are taken together. It can be noted from Table 12 that, highest classification performance is obtained using EbagT classifier compared to other classifiers used in our work.

## 5. Discussion

In the literature, the studies on detection of micro structure CAP phasic events are sparse and limited. On the other hand, a plethora of studies are available on identification of sleep macro-structures events including sleep stage scoring and identification of sleep disorders. Moreover, these handful of studies on CAP phase identification have used only healthy controls with few exceptions of Hartmann et al. [26] and Mendonca et al. [28], which have included either NFLE or SDB patients. In this proposed study, we have performed CAP phase identification using all 77 subjects comprising six types of sleep disordered patients having NFLE, SDB, narcolepsy, PLM, insomnia and RBD along with good sleepers.

We have also conducted the whole experiment taking 1-second EEG window in addition to normal 2 s window length. It can be observed from Table 4, Table 5, Table 6, Table 7, Table 8, Table 9, Table 10, Table 11 and Table 13 that, the phase classification performance is better for the epochs of 2 s than 1 s. Although the performance achieved using 1 s EEG epochs is inferior but the signal processing burden gets reduced on the system.

Our results reveal that for Healthy, Insomnia, and RBD subjects bipolar EEG analysis is better, which is in line with the observations made by Parrino et al. [22]. But, for Narcolepsy, NFLE and SBD subjects, monopolar EEG channel is found to be better. However, for accurate analysis, we believe that both channels are equally important. To the best of our knowledge, there is no clinical reason available regarding the superiority of channel (monopolar or bipolar). However, our results reveal that when both monopolar and bipolar EEG channel are used together, the performance of the model improved for sleep disorder as well for healthy good sleepers.

We have computed the microstructure details of subjects used in this study, like CAP rate, CAP time and NREM time (Table 14). It is evident from Table 14 that the average CAP rate is found to be 0.56 when all subjects (health+sleep disordered) are taken together. In the table, we have also mentioned the CAP rate for the six sleep disordered subjects and healthy controls when they are considered separately. It can be noticed from the table that the CAP rate of healthy subjects is lowest (0.41) and increased for sleep disordered subjects. The CAP rate for SDB patients is the highest (0.78) among all. RBD patients showed lower CAP rate (0.49) compared to other sleep disorders. Hence, CAP rate can be used as a measure to indicate the sleep quality. The CAP rate either increases or decreases sharply in sleep disorder cases. More precisely, the CAP rate increases in insomnia [52,53,54,55,56], apnea [57], PLM [58], NFLE [59], and depression [60,61]. The CAP rate decreases in conditions like narcolepsy [62], continuous positive airway pressure (CPAP) treatment in apnea [63,64,65,66] and neurodegenerative disorders, like Alzheimer’s disease [67].

Table 15 shows the comparison of our proposed method with other previously performed state-of-the-art studies. Various techniques have been employed by researchers for CAP phase detection. All the studies mentioned in the table have used CAP sleep database with majority of work being performed with EEG signals of healthy subjects.

Mendez et al. [25] used unbalanced data with 3963 Phase A events from only ten healthy adult subjects. They have used K-nearest neighbour (KNN) classifier and features including energy, sample entropy, standard deviation, Tsallis entropy and frequency band indices. They obtained an accuracy and sensitivity of around 80% and specificity of 70%. Navona et al. [24] have achieved an accuracy of 77% using EEG band descriptors and thresholding. Hartmann et al. [26] have used 16 healthy sleepers and 30 nocturnal frontal lobe epilepsy (NFLE) suffering subjects obtained from CAP sleep database. They achieved an accuracy of 82.42% for healthy subjects. The epoch length considered in their study is variable with a duration of 1–3 s. Dhok et al. [27] have used balanced data with 4653 occurrences of phase A and phase B each, from six healthy subjects of CAP sleep database for automated CAP phase classification. They used Wigner-Ville distribution based feature extraction and support vector machine (SVM) classifier to achieve classification accuracy of 72.35%. Mendonca et al. [28] have used time series analysis, Matrix of Lags and SVM classifier and obtained classification accuracy of 77% using ECG signals of 60 s duration. Recently, Loh et al. [68] developed a deep neural network (1D-CNN) model for CAP phase classification and obtained an accuracy of 73.64%. Mariani et al. [69] have observed that Hjorth actvity is a better descriptor for CAP A phases and helped to achieve better classification performance between phase A and phase B which is inline with the findings of our proposed study. It can be observed from the table that most of the studies have used only imbalanced data in which case the model developed may bias towards the majority class and cannot be considered as an ideal fit for a clinical application. The proposed study employed a balanced data to overcome bias, under fitting and over fitting problems. The proposed model attained ACA of 83% which is better than the most of the studies presented in Table 15.

The key attributes and benefits of our study are as follows:

- We have employed openly available CAP sleep database for easy reproducibility and to make it easy for other researchers to compare their work with this study.
- We have used only two EEG channels to reduce complexity and discomfort to patients. The simultaneous use of both C4-A1 and F4-C4 EEG channels improved the performance which is evident from our results.
- In addition to healthy subjects, we have also used subjects from six different CAP database cohorts like insomnia, narcolepsy, NFLE, PLM, RBD, and SDB.
- Unlike other studies, in the proposed method we have used less number of features which leads to lesser computational complexity.
- Along with 2-second EEG epochs, the simulations are also done with 1-second epochs.
- Taking into account the non-stationary nature of EEG signals, we have used wavelet-based Hjorth and entropy features which employed orthogonal filter bank. It can be observed from Table 4 and Table 16 that, the performance parameters for phase A and phase B classification of healthy subjects are lower if wavelet analysis is not employed.
- We have used balanced data in order to obtain robust classification.
- Our developed model is simple without involving much computational complexity and hence can be deployed in real time applications by medical practitioners.

It can be noted that, although the classification task considered is binary, the task is demanding due to high level of resemblance in characteristics of phases, which is clear from the results obtained by the state-of-the-art methods available. The classification accuracy in the range of 72–83% have been achieved using various techniques including few deep learning models also as shown in Table 15. Further, to examine the discriminating abilities of wavelet based features, we have performed the experiments without using wavelet decomposition and noticed high degradation in the performance (Table 16). The proposed method has yielded high classification results due to the use of highly discriminating nature of optimal wavelet based Hjorth features used by us to train the model. Our results demonstrated that if we do not use wavelet decomposition and use the Hjorth parameters of EEG directly, then the performance decreases. Similarly, if we use the wavelet decomposition and use some statistical features other than Hjorth parameters, again the performance degrades. On the other hand, when both optimal wavelet decomposition and Hjorth parameters are employed together, the optimal performance is obtained.

Presently, the CAP phases are identified by trained clinicians in sleep laboratories only. This process is cumbersome, stressful and time consuming. Hence, there is always a scope for a computer based automated approach. As discussed earlier, previous few studies have tried to develop a model for CAP characterization. Our method has achieved better performance for CAP phase identification. However, it should be tested using a large independent cohorts before implementing in a clinical application, this can be considered as one of the limitations of the proposed study. There is scope for further improvement in the results obtained using 1 s epochs. The future scope of this work includes the use of deep learning (DL) based techniques like convolutional neural network (CNN), LSTM and recurrent neural network (RNN) for an automated identification of CAP phases. Although, DL-based techniques perform better in many cases but for this problem, our proposed method worked better than rest of the reported works. Hartmann et al. [26] have already explored DL-based LSTM technique for automated detection of CAP phase A. Our results are found to be better because we have used highly discriminating wavelet-based features and optimal ensemble classifiers for the classification task. In future, it would be interesting to evaluate the performance of the proposed method for identifying sub-phases A1, A2, and A3 of the phase A.

## 6. Conclusions

The sleep-scoring is widely used in monitoring and analysis of sleep as well as identifying sleep disorders. The sleep macrostructure, represented by different sleep stages provide information regarding the neural activity and brain waves during sleep. However, the macrostructure sleep stages alone cannot provide information about the functional structure and stability of sleep. Besides, the shorter phasic events K complexes, delta-wave bursts, vertex waves, saw-tooth waves, sleep spindles and short-lasting arousals are also abundant in sleep. These events show certain patterns and represents the microstructure of sleep. CAP captures microstructure of the sleep and can be used to identify sleep instability. This paper presents an automated CAP characterization system using optimal wavelet-based features extracted from EEG signals. Our study aimed to reduce the diagnosis time of sleep by specialists. The main intention of the study is to identify the CAP phases of sleep disordered patients. We have utilized the entire CAP sleep database containing both sleep disordered patients and good sleepers. Our study presented the results obtained from healthy subjects, sleep disordered patients individually as well as all subjects combined. An optimal mean squared bandwidth minimized orthogonal filter bank is employed for the decomposition. The combination of highly discriminating wavelet entropy and Hjorth parameters coupled with optimally tuned ensemble bagged trees classier yielded a promising performance. The proposed model classified A and B phases of REM sleep. The model has yielded average classification accuracy of 84%, 83%, 81%, 78%, 77%, 76% and 72% for NFLE, healthy, SDB, narcolepsy, PLM, insomnia and RBD subjects, respectively in discriminating phases A and B using a balanced database. The best accuracy of 84% has been obtained for NFLE patients. However, the proposed model requires to be tested using a diverse and large data before clinical implementation. Our developed system is simple and fully computer-based, which can reduce the challenges faced by sleep specialists in scoring of CAP phases. In future, we aim to develop a model for the identification of subtypes A1, A2 and A3 of CAP phase A using DL based techniques like CNN, LSTM and RNN.

## Figures and Tables

**Table 1:** Demographic details of the subjects used in this study.

Subject Type	Subject Count	Gender		Age			Number of 2 s Epochs		
		Male	Female	Mean	Min	Max	Phase A	Phase B	Total
Healthy	6	2	4	32.00	23	37	4965	4965	9930
Insomnia	7	2	5	58.86	47	72	5664	5664	11,328
Narcolepsy	5	2	3	31.60	18	44	5043	5043	10,086
NFLE	27	13	14	30.04	16	67	36,618	36,618	73,236
PLM	9	6	3	54.44	40	62	10,029	10,029	20,058
RBD	22	19	3	70.73	58	82	19,827	19,827	39,654
SDB	1	1	0	78.00	78	78	834	834	1668
All Combined	77	45	32	48.01	16	82	82,980	82,980	165,960

**Table 2:** Details about Hjorth parameters used in this study [51].

Hjorth Parameter	Activity	Mobility (μ)	Complexity
Formula	$\sigma^{2}\left(x\left(t\right)\right)$	$\frac{\sigma (x^{\prime }\left(t\right))}{\sigma (x(t))}$	$\frac{\mu (x^{\prime }\left(t\right))}{\mu (x(t))}$
Description	It is the variance of the signal x(t). It represents total energy of the signal x(t).	It gives estimate of the mean frequency. It represents the proportion of standard deviation of the power spectrum.	It gives estimate of the bandwidth frequency. It represents change in frequency.

**Table 3:** *P*-value and rank for all 48 features computed using Kruskal-Wallis test.

Feature	Subband	C4-A1 Channel		F4-C4 Channel	
		*p*-Value	Rank	*p*-Value	Rank
Hjorth Activity	D1	0	35	0	6
	D2	0	2	0	25
	D3	$1.95\times 10^{-9}$	26	0	44
	D4	0	36	0	41
	D5	0	11	0	18
	A5	$7.80\times 10^{-28}$	29	0.073	21
Hjorth Mobility	D1	0	38	0	17
	D2	0	39	0	3
	D3	0	1	0	10
	D4	0	12	0	16
	D5	0	5	0	30
	A5	0	8	0	43
Hjorth Complexity	D1	0	27	0	31
	D2	0	4	0	40
	D3	0	33	0	13
	D4	0	19	0	48
	D5	$4.00\times 10^{-20}$	14	$5.04\times 10^{-31}$	24
	A5	$8.09\times 10^{-37}$	32	0	45
Wavelet Entropy	D1	0	9	0	37
	D2	0	20	0	46
	D3	0	28	0	47
	D4	0	15	0	23
	D5	0	42	0	22
	A5	0	7	0	34

**Table 4:** Classification performance for healthy subjects.

Predicted	Actual					
	C4-A1 Channel		F4-C4 Channel		Both Channels Combined	
	Phase A	Phase B	Phase A	Phase B	Phase A	Phase B
Phase A	77.30%	26.00%	78.10%	25.20%	83.30%	16.70%
Phase B	22.70%	74.00%	21.90%	74.80%	16.70%	83.30%
ACA, Pcn, Rcl	75.70%, 0.75, 0.77		76.40%, 0.76, 0.78		83.30%, 0.83, 0.83	
F1, κ, AUC	0.76, 0.53, 0.84		0.77, 0.51, 0.84		0.83, 0.67, 0.91	
Classifier	EBoosT		EBoosT		EBagT	

**Table 5:** Classification performance for insomnia subjects.

Predicted	Actual					
	C4-A1 Channel		F4-C4 Channel		Both Channels Combined	
	Phase A	Phase B	Phase A	Phase B	Phase A	Phase B
Phase A	67.20%	24.30%	67.50%	22.40%	71.70%	18.70%
Phase B	32.80%	75.70%	32.50%	77.60%	28.30%	81.30%
ACA, Pcn, Rcl	71.40%, 0.73, 0.67		72.50%, 0.75, 0.67		76.50%, 0.79, 0.72	
F1, κ, AUC	0.70, 0.45, 0.79		0.71, 0.43, 0.79		0.75, 0.53, 0.85	
Classifier	EBagT		EBoosT		EBagT	

**Table 6:** Classification performance for narcolepsy subjects.

Predicted	Actual					
	C4-A1 Channel		F4-C4 Channel		Both Channels Combined	
	Phase A	Phase B	Phase A	Phase B	Phase A	Phase B
Phase A	69.70%	26.10%	77.20%	37.50%	79.20%	24.80%
Phase B	30.30%	73.90%	22.80%	62.50%	20.80%	75.20%
ACA, Pcn, Rcl	71.80%, 0.73, 0.70		69.80%, 0.67, 0.77		77.20%, 0.76, 0.79	
F1, κ, AUC	0.71, 0.44, 0.78		0.72, 0.40, 0.77		0.78, 0.54, 0.86	
Classifier	EBoosT		EBoosT		EBoosT	

**Table 7:** Classification performance for NFLE subjects.

Predicted	Actual					
	C4-A1 Channel		F4-C4 Channel		Both Channels Combined	
	Phase A	Phase B	Phase A	Phase B	Phase A	Phase B
Phase A	75.00%	20.40%	74.50%	23.00%	82.80%	14.80%
Phase B	24.70%	79.60%	25.50%	77.00%	17.20%	85.20%
ACA, Pcn, Rcl	77.40%, 0.79, 0.75		75.80%, 0.76, 0.74		84.00%, 0.85, 0.83	
F1, κ, AUC	0.77, 0.55, 0.86		0.75, 0.51, 0.84		0.84, 0.68, 0.92	
Classifier	SVM		SVM		EBagT	

**Table 8:** Classification performance for PLM subjects.

Predicted	Actual					
	C4-A1 Channel		F4-C4 Channel		Both Channels Combined	
	Phase A	Phase B	Phase A	Phase B	Phase A	Phase B
Phase A	67.90%	24.20%	67.60%	26.20%	72.80%	19.00%
Phase B	32.10%	75.80%	32.40%	73.80%	27.20%	81.00%
ACA, Pcn, Rcl	71.90%, 0.74, 0.70		70.70%, 0.72, 0.68		76.90%, 0.79, 0.73	
F1, κ, AUC	0.71, 0.44, 0.79		0.70, 0.41, 0.78		0.76, 0.54, 0.85	
Classifier	EBoosT		EBoosT		EBagT	

**Table 9:** Classification performance for RBD subjects.

Predicted	Actual					
	C4-A1 Channel		F4-C4 Channel		Both Channels Combined	
	Phase A	Phase B	Phase A	Phase B	Phase A	Phase B
Phase A	60.20%	28.20%	62.70%	27.80%	67.20%	22.80%
Phase B	39.80%	71.80%	37.30%	72.20%	32.80%	77.20%
ACA, Pcn, Rcl	66.00%, 0.68, 0.60		67.45%, 0.69, 0.63		72.20%, 0.75, 0.67	
F1, κ, AUC	0.64, 0.32, 0.72		0.66, 0.35, 0.74		0.71, 0.44, 0.80	
Classifier	EBagT		EBagT		EBagT	

**Table 10:** Classification performance for SDB subjects.

Predicted	Actual					
	C4-A1 Channel		F4-C4 Channel		Both Channels Combined	
	Phase A	Phase B	Phase A	Phase B	Phase A	Phase B
Phase A	70.50%	20.60%	71.00%	21.10%	80.30%	17.40%
Phase B	29.50%	79.40%	29.00%	78.90%	19.70%	82.60%
ACA, Pcn, Rcl	74.95%, 0.77, 0.70		74.95%, 0.77, 0.71		81.45%, 0.82, 0.80	
F1, κ, AUC	0.74, 0.50, 0.82		0.74, 0.50, 0.82		0.81, 0.63, 0.89	
Classifier	EBagT		SVM		EBagT	

**Table 11:** Classification performance for all subjects combined.

Predicted	Actual					
	C4-A1 Channel		F4-C4 Channel		Both Channels Combined	
	Phase A	Phase B	Phase A	Phase B	Phase A	Phase B
Phase A	67.60%	24.70%	68.10%	26.20%	74.30%	18.20%
Phase B	32.40%	75.30%	31.90%	73.80%	25.70%	81.80%
ACA, Pcn, Rcl	71.45%, 0.73, 0.68		70.95%, 0.72, 0.68		78.00%, 0.80, 0.74	
F1, κ, AUC	0.70, 0.42, 0.79		0.70, 0.42, 0.78		0.77, 0.56, 0.86	
Classifier	EBagT		EBagT		EBagT	

**Table 12:** Summary of performances obtained by various classifiers using features extracted from both EEG channels of 77 subjects with ten-fold CV strategy. The bold font style represents maximum ACA.

Classifier	Performance Parameters		
	ACA (%)	F1 Score	κ
Fine Tree	75.93	0.7681	0.52
Logistic Regression	66.88	0.6947	0.34
SVM	74.01	0.7596	0.48
KNN	70.68	0.7376	0.41
EBagT	**78.00**	0.7752	0.56

**Table 13:** Performance parameters obtained for CAP Phase A detection in different types of sleep disorder subjects using both EEG channels for 1-sec epoch duration.

Subject	C4-A1 Channel			F4-C4 Channel			C4-A1 + F4-C4 Channels		
	ACA	F1-Score	κ	ACA	F1-Score	Kappa	ACA	F1-Score	κ
Healthy	0.72	0.72	0.44	0.72	0.72	0.44	0.79	0.79	0.58
Insomnia	0.67	0.66	0.35	0.69	0.67	0.38	0.74	0.72	0.48
Narcolepsy	0.68	0.67	0.36	0.68	0.70	0.36	0.74	0.74	0.48
NFLE	0.73	0.73	0.47	0.72	0.72	0.47	0.75	0.73	0.49
PLM	0.67	0.66	0.35	0.67	0.66	0.35	0.73	0.71	0.46
RBD	0.62	0.60	0.25	0.64	0.62	0.28	0.67	0.65	0.33
SDB	0.70	0.70	0.41	0.71	0.71	0.41	0.76	0.77	0.53
All	0.68	0.67	0.36	0.68	0.67	0.36	0.74	0.72	0.48
Classifier	EBoosT						EBagT		

**Table 14:** Details of sleep microstructure for subjects used in this study.

Subject Type	Duration	NREM Time	CAP Time	CAP Rate
Healthy	505.33	350.83	144.50	0.41
Insomnia	575.71	269.00	135.86	0.51
Narcolepsy	494.40	299.20	159.40	0.53
NFLE	505.81	353.93	223.22	0.63
PLM	431.33	282.00	186.44	0.68
RBD	514.14	315.68	152.50	0.49
SDB	396.00	283.00	221.00	0.78
All Combined (Avg)	503.64	322.16	180.47	0.56

**Table 15:** Comparison our study with state-of-the-art techniques for CAP phase identification using healthy subjects.

Study	Method	Subjects	Epoch Length	ACA (%)	Recall	F1-Score
Mendez et al. [25]	KNN classifier	10	2 s	80.00	0.70	0.75
Navona et al. [24]	Band descriptors and Thresholding	10	2 s	77.00	0.84	0.87
Hartmann et al. [26]	Variable LSTM Network, Hjorth activity, Band power, Teager energy operator, Shannon entropy	46	1–3 s	82.42	0.75	0.79
Dhok et al. [27]	Wigner-Ville based Renyi Entropy, SVM	6	2 s	72.35	0.77	0.73
Mendonca et al. [28] (ECG)	Time series analysis, Matrix of Lags, SVM	13	60 s	77.00	0.71	0.76
Loh et al. [68]	Deep neural network (1D-CNN)	6	2 s	73.64	0.80	0.75
Proposed study	Optimal wavelet based Hjorth and entropy features	77	2 s	83.30	0.83	0.83

**Table 16:** CAP phase classification results obtained for healthy subjects without wavelet decomposition.

Channel	Performance Parameters					
	Accuracy (%)	Precision	Recall	F1-Score	κ	AUC
C4-A1	73.36	0.7413	0.7201	0.7306	0.4717	0.81
F4-C4	73.74	0.7484	0.7152	0.7314	0.4747	0.81
C4-A1 + F4-C4	76.86	0.7177	0.7630	0.7403	0.5171	0.83

## Data Availability

Data used in this work is open-source and publicly available on PhysioNet (https://physionet.org/content/capslpdb/1.0.0/, accessed on 29 July 2021).

## References

[1] Iber C. (2007). The AASM manual for the scoring of sleep and associated events: Rules. Terminol. Tech. Specif..

[2] Kim J., Lee J., Shin M. (2017). Sleep stage classification based on noise-reduced fractal property of heart rate variability. Procedia Comput. Sci..

[3] Sharma M., Patel S., Choudhary S., Acharya U.R. (2019). Automated Detection of Sleep Stages Using Energy-Localized Orthogonal Wavelet Filter Banks. Arab. J. Sci. Eng..

[4] Sharma M., Tiwari J., Acharya U.R. (2021). Automatic Sleep-Stage Scoring in Healthy and Sleep Disorder Patients Using Optimal Wavelet Filter Bank Technique with EEG Signals. Int. J. Environ. Res. Public Health.

[5] Sharma M., Goyal D., Achuth P., Acharya U.R. (2018). An accurate sleep stages classification system using a new class of optimally time-frequency localized three-band wavelet filter bank. Comput. Biol. Med..

[6] Fiorillo L., Puiatti A., Papandrea M., Ratti P.L., Favaro P., Roth C., Bargiotas P., Bassetti C., Faraci F. (2019). Automated sleep scoring: A review of the latest approaches. Sleep Med. Rev..

[7] Alickovic E., Subasi A. (2018). Ensemble SVM method for automatic sleep stage classification. IEEE Trans. Instrum. Meas..

[8] Faust O., Razaghi H., Barika R., Ciaccio E.J., Acharya U.R. (2019). A review of automated sleep stage scoring based on physiological signals for the new millennia. Comput. Methods Programs Biomed..

[9] Hassan A.R., Bhuiyan M.I.H. (2017). Automated identification of sleep states from EEG signals by means of ensemble empirical mode decomposition and random under sampling boosting. Comput. Methods Programs Biomed..

[10] Loh H., Ooi C., Vicnesh J., Oh S.L., Faust O., Gertych A., Acharya U.R. (2020). Automated Detection of Sleep Stages Using Deep Learning Techniques: A Systematic Review of the Last Decade (2010–2020). Appl. Sci..

[11] Yildirim O., Baloglu U.B., Acharya U.R. (2019). A Deep Learning Model for Automated Sleep Stages Classification Using PSG Signals. Int. J. Environ. Res. Public Health.

[12] Zhang J., Wu Y. (2017). A new method for automatic sleep stage classification. IEEE Trans. Biomed. Circuits Syst..

[13] Terzano M.G., Parrino L. (2000). Origin and significance of the cyclic alternating pattern (CAP). Sleep Med. Rev..

[14] Hartmann S., Bruni O., Ferri R., Redline S., Baumert M. (2020). Characterization of cyclic alternating pattern during sleep in older men and women using large population studies. Sleep.

[15] Terzano M.G., Parrino L., Sherieri A., Chervin R., Chokroverty S., Guilleminault C., Hirshkowitz M., Mahowald M., Moldofsky H., Rosa A. (2001). Atlas, rules, and recording techniques for the scoring of cyclic alternating pattern (CAP) in human sleep. Sleep Med..

[16] Terzano M., Parrino L. (1994). Erratum: Clinical applications of cyclic alternating patterns. Physiol. Behav..

[17] Ferini-Strambi L., Bianchi A., Zucconi M., Oldani A., Castronovo V., Smirne S. (2000). The impact of cyclic alternating pattern on heart rate variability during sleep in healthy young adults. Clin. Neurophysiol..

[18] Fischgold H., Mathis P. (1959). Obnubilations, comas et stupeurs. Etudes électroéncephalographiques. Electroencephalogr. Clin. Neurophysiol..

[19] INGVAR D.H., LUNDBERG N. (1961). Paroxysmal symptoms in intracranial hypertension, studied with ventricular fluid pressure recording and electroencephalography. Brain.

[20] Ferri R., Bruni O., Miano S., Plazzi G., Spruyt K., Gozal D., Terzano M.G. (2006). The time structure of the cyclic alternating pattern during sleep. Sleep.

[21] Terzano M., Mancia D., Salati M., Costani G., Decembrino A., Parrino L. (1985). The cyclic alternating pattern as a physiologic component of normal NREM sleep. Sleep.

[22] Parrino L., Ferri R., Bruni O., Terzano M.G. (2012). Cyclic alternating pattern (CAP): The marker of sleep instability. Sleep Med. Rev..

[23] Smerieri A., Parrino L., Agosti M., Ferri R., Terzano M.G. (2007). Cyclic alternating pattern sequences and non-cyclic alternating pattern periods in human sleep. Clin. Neurophysiol..

[24] Navona C., Barcaro U., Bonanni E., Di Martino F., Maestri M., Murri L. (2002). An automatic method for the recognition and classification of the A-phases of the cyclic alternating pattern. Clin. Neurophysiol..

[25] Mendez M.O., Chouvarda I., Alba A., Bianchi A.M., Grassi A., Arce-Santana E., Milioli G., Terzano M.G., Parrino L. (2016). Analysis of A-phase transitions during the cyclic alternating pattern under normal sleep. Med. Biol. Eng. Comput..

[26] Hartmann S., Baumert M. (2019). Automatic a-phase detection of cyclic alternating patterns in sleep using dynamic temporal information. IEEE Trans. Neural Syst. Rehabil. Eng..

[27] Dhok S., Pimpalkhute V., Chandurkar A., Bhurane A.A., Sharma M., Acharya U.R. (2020). Automated phase classification in cyclic alternating patterns in sleep stages using Wigner–Ville distribution based features. Comput. Biol. Med..

[28] Mendonça F., Mostafa S.S., Morgado-Dias F., Ravelo-García A.G. (2020). Matrix of Lags: A tool for analysis of multiple dependent time series applied for CAP scoring. Comput. Methods Programs Biomed..

[29] Rajput J.S., Sharma M., Tan R.S., Acharya U.R. (2020). Automated detection of severity of hypertension ECG signals using an optimal bi-orthogonal wavelet filter bank. Comput. Biol. Med..

[30] Jiang X., Bian G.B., Tian Z. (2019). Removal of artifacts from EEG signals: A review. Sensors.

[31] Lai C.Q., Ibrahim H., Abdullah M.Z., Abdullah J.M., Suandi S.A., Azman A. Artifacts and noise removal for electroencephalogram (EEG): A literature review. Proceedings of the 2018 IEEE Symposium on Computer Applications & Industrial Electronics (ISCAIE).

[32] Bhati D., Sharma M., Pachori R.B., Nair S.S., Gadre V.M. (2016). Design of Time–Frequency Optimal Three-Band Wavelet Filter Banks with Unit Sobolev Regularity Using Frequency Domain Sampling. Circuits Syst. Signal Process..

[33] Sharma M., Dhere A., Pachori R.B., Acharya U.R. (2017). An automatic detection of focal EEG signals using new class of time–frequency localized orthogonal wavelet filter banks. Knowl.-Based Syst..

[34] Sharma M., Dhere A., Pachori R.B., Gadre V.M. (2017). Optimal duration-bandwidth localized antisymmetric biorthogonal wavelet filters. Signal Process..

[35] Sharma M., Tan R.S., Acharya U.R. (2019). Detection of shockable ventricular arrhythmia using optimal orthogonal wavelet filters. Neural Comput. Appl..

[36] Sharma M., Acharya U.R. (2021). Automated detection of schizophrenia using optimal wavelet-based *l*_1_ norm features extracted from single-channel EEG. Cogn. Neurodynamics.

[37] Sharma M., Deb D., Acharya U.R. (2018). A novel three-band orthogonal wavelet filter bank method for an automated identification of alcoholic EEG signals. Appl. Intell..

[38] Sharma M., Acharya U.R. (2018). Analysis of knee-joint vibroarthographic signals using bandwidth-duration localized three-channel filter bank. Comput. Electr. Eng..

[39] Sharma M., Acharya U.R. (2019). A new method to identify coronary artery disease with ECG signals and time-Frequency concentrated antisymmetric biorthogonal wavelet filter bank. Pattern Recognit. Lett..

[40] Luo Z., Tay D., Lai X., Lin Z. Design of Orthogonal Wavelet Filters with Minimum RMS Bandwidth Using A Symbolic Approach. Proceedings of the 2021 IEEE International Symposium on Circuits and Systems (ISCAS).

[41] Sharma M., Bhurane A.A., Acharya U.R. (2018). MMSFL-OWFB: A novel class of orthogonal wavelet filters for epileptic seizure detection. Knowl.-Based Syst..

[42] Sharma M., Tan R.S., Acharya U.R. (2019). Automated heartbeat classification and detection of arrhythmia using optimal orthogonal wavelet filters. Informatics Med. Unlocked.

[43] Sharma M., Vanmali A.V., Gadre V.M. (2013). Construction of wavelets: Principles and practices. Wavelets and Fractals in Earth System Sciences.

[44] Sharma M., Kolte R., Patwardhan P., Gadre V. (2010). Time-frequency localization optimized biorthogonal wavelets. Int. Conf. Signal Process. Comm. (SPCOM).

[45] Sharma M., Bhati D., Pillai S., Pachori R.B., Gadre V.M. (2016). Design of Time–Frequency Localized Filter Banks: Transforming Non-convex Problem into Convex Via Semidefinite Relaxation Technique. Circuits Syst. Signal Process..

[46] Sharma M., Singh T., Bhati D., Gadre V. Design of two-channel linear phase biorthogonal wavelet filter banks via convex optimization. Proceedings of the 2014 International Conference on Signal Processing and Communications (SPCOM).

[47] Sharma M., Gadre V.M., Porwal S. (2015). An Eigenfilter-Based Approach to the Design of Time-Frequency Localization Optimized Two-Channel Linear Phase Biorthogonal Filter Banks. Circ. Syst. Signal Process..

[48] Bhurane A., Dhok S., Sharma M., Rajamanickam Y., M M., Acharya U.R. (2019). Diagnosis of Parkinson’s Disease from EEG signals using Linear and Self-Similarity features. Expert Syst..

[49] Upadhyay A., Sharma M., Pachori R.B. (2017). Determination of instantaneous fundamental frequency of speech signals using variational mode decomposition. Comput. Electr. Eng..

[50] Coifman R.R., Wickerhauser M.V. (1992). Entropy-based algorithms for best basis selection. IEEE Trans. Inf. Theory.

[51] Hjorth B. (1970). EEG analysis based on time domain properties. Electroencephalogr. Clin. Neurophysiol..

[52] Terzano M., Parrino L. (1992). Evaluation of EEG Cyclic Alternating Pattern during Sleep in Insomniacs and Controls under Placebo and Acute Treatment with Zolpidem. Sleep.

[53] Parrino L., Spaggiari M., Boselli M., Giovanni G., Terzano M. (1994). Clinical and polysomnographic effects of trazodone CR in chronic insomnia associated with dysthymia. Psychopharmacology.

[54] Parrino L., Milioli G., Paolis F., Grassi A., Terzano M. (2009). Paradoxical insomnia: The role of CAP and arousals in sleep misperception. Sleep Med..

[55] Sharma M., Patel V., Acharya U.R. (2021). Automated identification of insomnia using optimal bi-orthogonal wavelet transform technique with single-channel EEG signals. Knowl.-Based Syst..

[56] Sharma M., Dhiman H.S., Acharya U.R. (2021). Automatic identification of insomnia using optimal antisymmetric biorthogonal wavelet filter bank with ECG signals. Comput. Biol. Med..

[57] Terzano M., Parrino L., Boselli M., Spaggiari M., Giovanni G. (1996). Polysomnographic Analysis of Arousal Responses in Obstructive Sleep Apnea Syndrome by Means of the Cyclic Alternating Pattern. J. Clin. Neurophysiol. Off. Publ. Am. Electroencephalogr. Soc..

[58] Parrino L., Boselli M., Buccino G., Spaggiari M., Giovanni G., Terzano M. (1996). The Cyclic Alternating Pattern Plays a Gate-Control on Periodic Limb Movements During Non-Rapid Eye Movement Sleep. J. Clin. Neurophysiol. Off. Publ. Am. Electroencephalogr. Soc..

[59] Zucconi M., Oldani A., Smirne S., Ferini-Strambi L. (2000). The Macrostructure and Microstructure of Sleep in Patients With Autosomal Dominant Nocturnal Frontal Lobe Epilepsy. J. Clin. Neurophysiol. Off. Publ. Am. Electroencephalogr. Soc..

[60] Farina B., Della Marca G., Grochocinski V., Mazza M., Buysse D., di Giannantonio M., Mennuni G., Risio S., Kupfer D., Frank E. (2004). Microstructure of sleep in depressed patients according to the cyclic alternating pattern. J. Affect. Disord..

[61] Sharma M., Achuth P., Deb D., Puthankattil S.D., Acharya U.R. (2018). An Automated Diagnosis of Depression Using Three-Channel Bandwidth-Duration Localized Wavelet Filter Bank with EEG Signals. Cogn. Syst. Res..

[62] Terzano M., Smerieri A., Del Felice A., Giglia F., Palomba V., Parrino L. (2007). Cyclic alternating pattern (CAP) alterations in narcolepsy. Sleep Med..

[63] Parrino L., Smerieri A., Boselli M., Spaggiari M., Terzano M. (2000). Sleep reactivity during acute nasal CPAP in obstructive sleep apnea syndrome. Neurology.

[64] Sharma M., Kumbhani D., Yadav A., Acharya U.R. (2021). Automated Sleep apnea detection using optimal duration-frequency concentrated wavelet-based features of pulse oximetry signals. Appl. Intell..

[65] Sharma M., Agarwal S., Acharya U.R. (2018). Application of an optimal class of antisymmetric wavelet filter banks for obstructive sleep apnea diagnosis using ECG signals. Comput. Biol. Med..

[66] Sharma M., Raval M., Acharya U.R. (2019). A new approach to identify obstructive sleep apnea using an optimal orthogonal wavelet filter bank with ECG signals. Informatics Med. Unlocked.

[67] Maestri M., Carnicelli L., Tognoni G., Coscio E., Giorgi F., Volpi L., Economou N.T., Ktonas P., Ferri R., Bonuccelli U. (2015). Non–rapid eye movement sleep instability in mild cognitive impairment: A pilot study. Sleep Med..

[68] Loh H., Ooi C., Dhok S., Sharma M., Bhurane A., Acharya U.R. (2021). Automated detection of cyclic alternating pattern and classification of sleep stages using deep neural network. Appl. Intell..

[69] Mariani S., Manfredini E., Rosso V., Mendez M.O., Bianchi A.M., Matteucci M., Terzano M.G., Cerutti S., Parrino L. (2011). Characterization of A phases during the cyclic alternating pattern of sleep. Clin. Neurophysiol..

